# Co-occurrence Patterns of Intimate Partner Violence

**Published:** 2021

**Authors:** Ahmet Hacıaliefendioğlu, Serhan Yılmaz, Mehmet Koyutürk, Günnur Karakurt

**Affiliations:** 1 Department of Computer and Data Sciences, Case Western Reserve University, Cleveland, OH, USA; 2 Center for Proteomics and Bioinformatics, Case Western Reserve University, Cleveland, OH, USA; 3 Department of Psychiatry, Case Western Reserve University, Cleveland, OH, USA; 4 University Hospitals, Cleveland, OH, USA

**Keywords:** Intimate partner violence, psychological aggression, physical violence, sexual violence, micro-aggression, co-occurrence, network analysis, clustering, data visualization

## Abstract

Intimate partner violence (IPV) is an important social and public health problem, affecting millions of women worldwide. Violence in a relationship can occur in multiple ways, including physical violence, psychological aggression, and sexual violence. In this study, utilizing data from the National Intimate Partner and Sexual Violence Survey (NISVS), we comprehensively investigate the interplay between physical, psychological, and sexual violence, in terms of their co-occurrence patterns, their relation to trauma symptoms and overall health of victims. For this purpose, we perform network analysis and develop a visualization technique that enables in-depth navigation of the three-dimensional (physical, psychological, sexual) space of violence. Our findings show that physical violence tends to significantly co-occur with psychological abuse, and violence intensifies when both are present. We also find that sexual violence tends to overlap less with other types of violence, particularly with physical violence. Milder forms of psychological abuse are prominent in the population and seem to represent a separate type of abuse (micro-aggression) in terms of its occurrence patterns. Finally, we observe that trauma symptoms and health problems tend to be reported more by survivors at the presence of intense psychological aggression. Our findings can be useful in developing treatments that target different patterns of IPV.

## 1. Introduction

Intimate partner violence (IPV), also commonly referred to as domestic violence, is a significant public health issue that adversely affects the well-being of millions of women across the world. IPV is often defined as physical, sexual, and psychological aggression by a current or former intimate partner. According to CDC data, during their lifetime, one in every four women experience severe forms of physical violence.^1^ Breiding *et al.*^1^ define physical violence as using physical force with the intent to harm, inflict injury or cause death. Physical violence encompasses behaviors such as pushing, punching, kicking and using weapons.^2^ Psychological aggression is defined as using communication, both verbal and non-verbal, with intent to mentally and emotionally harm another person. They also include exerting control into their definition.^1^ Psychological aggression encompasses explosive anger, coercive control, degradation and isolation.^3,4^ The definition of sexual violence includes any sexual acts either committed or attempted without the informed consent of the victim and/or despite their refusal.^1^ Sexual violence encompasses but is not limited to intentional unwanted sexual touching, pressuring for sex, and forced penetration.^1,2^ The intensity of IPV cases range in severity from executing threats to committing homicide.^5^

Harmful effects of IPV on the physical health of women are often linked to acute injuries including bruises, lacerations, fractions, as well as chronic conditions including chronic pain syndrome, hypertension, and fibromyalgia.^5^ IPV is also detrimental to sexual health and is frequently linked with sexually transmitted infections and urinary tract infections.^6^ Furthermore, IPV’s harmful effects on mental health are often associated with depression, anxiety, post-traumatic stress disorders, excessive stress, and suicidality^4,7^.^8^

The co-occurrence of multiple types of violence is also common.^9^ Different types of violence can co-occur with varying ranges of intensity in a relationship^9^.^10^ Indeed, past research indicated high positive correlation with psychological and physical abuse.^11^ Identifying patterns of different types of IPV that are simultaneously occurring in the relationships can help immensely with treatment efforts.^12^ However, elucidation of these complex co-occurrence patterns require comprehensive computational analyses on large scale data. In this paper, capitalizing on the availability of data from the National Intimate Partner and Sexual Violence Survey (NISVS), we aim to comprehensively characterize the co-occurrence patterns of IPV.

NISVS surveyed thousands of women in the United States to collect comprehensive data on the manifestation of different types of violence. While these large-scale data have been useful in assessing the prevalence and intensity of different types of violence, little is known on the interplay between these different types. Here, we develop a comprehensive computational framework to systematically characterize the interplay between different types of violence. Our computational framework and contributions include the following components:

1. Using contingency analysis, we comprehensively quantify the overlap between four different types of violence (also including micro-aggression (MA) in addition to the other three types that are explicitly measured, as our analysis suggests that MA comprises an individual type of violence in terms of its prevalence and occurrence patterns).
2. Using network analysis, we investigate the co-occurrence of individual violence items and identify the items that are central to each violence type and characteristic of the interplay between different violence types.
3. We develop a radial visualization technique that quantifies the intensity and the type of IPV (reported by a survivor), which allows elaborate visualization of the interplay between different violence types and subgroups, as well as the projection of other variables (trauma symptoms, health problems) to the space defined by violence type and intensity.
4. Using clustering, we identify subgroups of survivors who are similar in terms of their reported violence and assess how the resulting subgroups align with violence types.

## 2. Materials and Methods

### 2.1. Description of Data and Pre-Processing

Data from the National Intimate Partner and Sexual Violence Survey (NISVS) is utilized in this study.^13^ This data was obtained through phone surveys of households across the United States. Randomly selected households were sent letters indicating they would be contacted for an interview. Overall, 16507 participants completed the interviews through the end.

NISVS is specifically designed to measure various characteristics related to relationship demographics, IPV and their adverse effects on health. The 39 items measuring the type, frequency and intensity of IPV are listed in Table 1. These items ask how many times the perpetrator did a specific action in the past year and answers are rated by the survivor in a scale of 0 (never), 1 (ten time), 2 (two to ten times), 3 (eleven to fifty times), and 4 (more than fifty times). We use these reported numbers directly in our analyses as an approximation to log-transformed frequencies of occurrence.

The items in the violence questionnaire are grouped into three violence types: (i) Physical violence (PV, 12 items), (ii) Sexual violence (SV, 22 items), and (iii) Psychological aggression (PA, 5 items). As we discuss in Section 3, we move some items between violence types. Based on occurrence patterns, we also separate one item in the PA group as a separate violence type. Namely, we observe that the item “called you names like ugly, fat, crazy, or stupid” appears too frequently and lies as an outlier in the principal component space (Figure 2(b)). To facilitate thorough analysis of this frequent item with its own occurrence pattern, we separate this item as fourth violence type termed micro-aggression (MA), This results in the following number of items per violence type: 10 items for PV, 23 items for SV, 5 items for PA, and 1 item for MA. In addition, the dataset includes 16 items measuring health problems (including asthma, diabetes, irritable bowel syndrome, high blood pressure, frequent headaches, chronic pain, difficulty sleeping, stress, perceived physical and mental health) as well as items measuring trauma symptoms (including concern for safety, fear, having nightmares, and desire to avoid remembering).

#### Filtering the survivors.

Since the study is performed on randomly selected households, most of the participants did not report any IPV. We also exclude instances where the perpetrator is not an intimate partner. Among the 16,507 participants who completed the survey, 873 of them reported at least one incidence of IPV in the past year (i.e., responded 1–4 to at least one of the 39 items in the survey). We focus on these 873 survivors in this study.

#### Data matrix and the computation of scores for violence types.

Filtering results in a 873×39 data matrix *R* of survivors vs. items, where *R*(*i, j*) ∈ {0, 1, 2, 3, 4} represents the response of survivor *i* to item *j*. We systematically analyze this data matrix from the perspective of survivors, as well as items. For this purpose, we call each row of this matrix a *survivor profile* and each column of this matrix an *item profile*. To assess the intensity of violence for each type, for each survivor, we compute an aggregate score averaging the responses of all items in the respective subscale. These scores, denoted *s*_*PV*_ (*i*), *s*_*PA*_(*i*), *s*_*SV*_ (*i*), and *s*_*MA*_(*i*) for survivor *i*, provide a summary statistic of the intensity of a particular violence type for the survivor.

### 2.2. Co-Occurrence of Violence Types

Here, we aim to assess whether violence types have a strong association with each other. For this purpose, for each violence type (PA, PV, SV, and MA), we identify the set of survivors who report a “high” level of violence in that category. To identify “high” levels of violence in a category, we use the population mean of *s*_*T*_ as a threshold. Namely, if $s_{T}(i)>{\bar{s}}_{T}$ for participant *i*, we consider that participant *i* reports high violence in category *T*, where ${\bar{s}}_{T}=\sum_{i=1}^{n}s(i)/n$. We denote the number of participants who report “high” levels of violence in type *T* according to this threshold as $n_{H}(T)=\left|\left\{i:s_{T}(i)>{\bar{s}}_{T}\right\}\right|$. While we report results according to population mean as the threshold, the results we obtain with different thresholds (including a threshold of zero, i.e., existence of violence or one or two standard deviation(s) above mean) are similar.

We assess the pairwise co-occurrence between two violence type *T* and *T*′ as $n_{HH}\left(T,T^{\prime }\right)=|\left\{i:s_{T}(i)>{\bar{s}}_{T}\;\text{and}\;s_{T}^{\prime }(i)>{\bar{s}}_{T}^{\prime }\right\}|$, i.e., the number of survivors who report both *T* and *T*′ above population mean. To provide a baseline for expected co-occurrence, we compute the expected number of overlaps based on the assumption that the two violence types are independent, i.e., *E*[*N*_*HH*_(*T, T*′)] = *n*_*H*_(*T*)*n*_*H*_(*T*′)*/n*, where *N*_*HH*_(*T, T*′) denotes the random variable that represents the co-occurrence of *T* and *T*′ (with observed value *n*_*HH*_(*T, T*′)).

To quantify the magnitude of the co-occurrence between *T* and *T*′, we use odds ratios:^14^

(1) $$OR\left(T,T^{\prime }\right)=\frac{n_{HH}\left(T,T^{\prime }\right)n_{LL}\left(T,T^{\prime }\right)}{n_{HL}\left(T,T^{\prime }\right)n_{LH}\left(T,T^{\prime }\right)}.$$

Here, $n_{LL}\left(T,T^{\prime }\right)=|\left\{i:s_{T}(i)\leq {\bar{s}}_{T}\;\text{and}\;s_{T}^{\prime }(i)\leq {\bar{s}}_{T}^{\prime }\right\}|$ denotes the number of survivors who report “low” violence for both types *T* and *T*′. *n*_*HL*_(*T, T*′) and *n*_*HL*_(*T, T*′) are defined similarly as respectively “high *T*”/“low *T*” and “low *T*”/“high *T*”. To assess the statistical significance of the odds ratios, we compute 95% confidence intervals as follows:

(2) $$\begin{array}{l} SE\left(T,T^{\prime }\right)={\left(\frac{1}{n_{HH}\left(T,T^{\prime }\right)}+\frac{1}{n_{LL}\left(T,T^{\prime }\right)}+\frac{1}{n_{HL}\left(T,T^{\prime }\right)}+\frac{1}{n_{LH}\left(T,T^{\prime }\right)}\right)}^{1/2}\\ OR_{\max}\left(T,T^{\prime }\right)=OR\left(T,T^{\prime }\right)e^{SE\left(T,T^{\prime }\right)},\;OR_{\min}\left(T,T^{\prime }\right)=OR\left(T,T^{\prime }\right)/e^{SE\left(T,T^{\prime }\right)}. \end{array}$$

### 2.3. Co-Occurrence Network of Individual Violence Items

To obtain the co-occurrence network between individual violence items, we first compute their Pearson correlation between the item profiles in a pairwise manner. We then construct a network by putting an edge between two items if they exhibit positive correlation greater than a given threshold (we use 0.2 in the results presented Section 3).

### 2.4. Radial Visualization

**Fig. 1:**
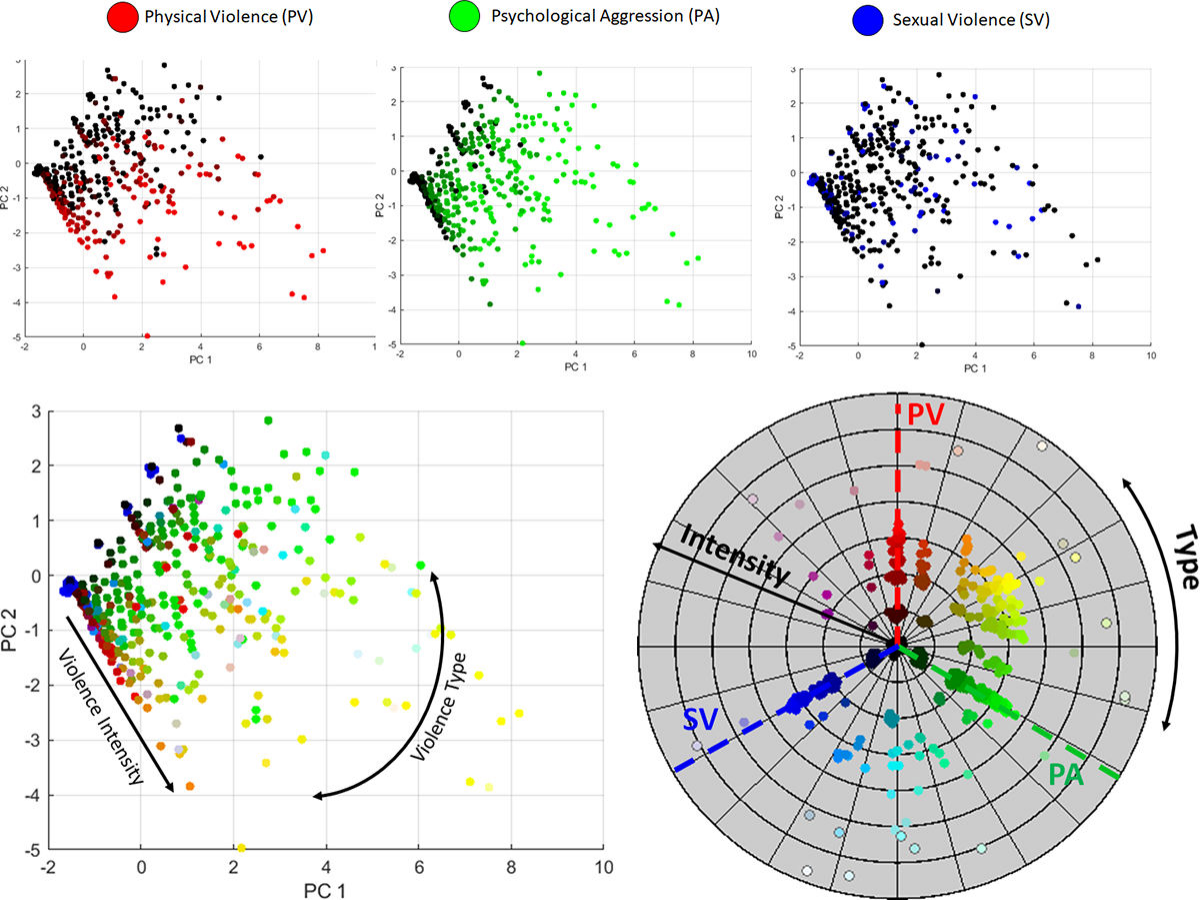
Using radial projection to visualize survivors in the three-dimensional space of violence types. (Top) Distribution of physical violence (PV), psychological aggression (PA), sexual violence (SV) scores in the plane of first two principal components. Coloring indicates the intensity of the corresponding violence type (PV, PA or SV). (Bottom Left) Distribution of PV, PA and SV scores in the plane of the first two principal components. Coloring is done according to PV, PA and SV scores: Red component: PV. Green component: PA, Blue Component: SV. As it can be seen, the first two principal components reflect the violence intensity as well as the violence type. (Bottom Right) Projection of the survivors to the radial space of violence type vs. violence intensity.

To investigate the relationship between violence types, we apply principal component analysis (PCA) to map survivors to the 2-dimensional PCA space as shown in Figure 1. In the top panel of this figure, the survivors are colored according to each violence type separately. To color the survivors according to violence type scores, we first use *rank normalization* to normalize the scores into the [0, 1] range. For this purpose, separately for each violence type *T*, we sort the survivors according to their *s*_*T*_ scores. Then, for each survivor *i*, we take the percentile of that survivor according to this ranking as their rank normalized score *r*_*T*_ (*i*).

**Fig. 2:**
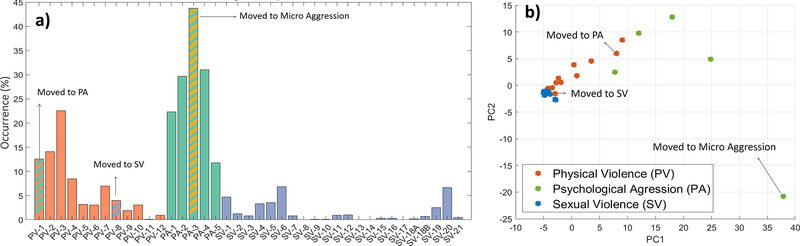
Response rate and principal component analysis of the items in the questionnaire. a) Reporting frequencies of the questionnaire items among the 873 survey survivors who report an incidence of intimate partner violence. Items are grouped and colored according to their scales: Physical Violence (PV), Psychological Aggression (PA), and Sexual Violence (SV). b) The projection of items projected on the space induced by the first two principal components. Each item is colored according to their scales. The items that were moved to another scale are marked.

The brightness of the R/G/B channel for each survivor indicates is set to be proportional to this rank-normalized score for respectively PV, PA, and SV. As seen in the figure, survivors with high PV score are typically clustered on the bottom side of the PCA plane and the survivors with high PA score are typically clustered on top right side of the PCA plane. Survivors with high SV scores do not appear to be clustered. This is not surprising since SV items are given little weight by the principal components (Figure 2a) due to the relative rarity of SV.

When we integrate the RGB values to visualize all violence types at once, we obtain the plot in the bottom left panel of Figure 1, leading to two interesting observations: (i) The intensity of the violence (as well as the brightness of the color) typically increases as the distance from the center in the PCA plane (middle left corner) increase, and (ii) The type of the violence (as well as the hue of the color) changes depending on the angle of the surrounding arc. This means that the principal component analysis essentially captures these two inherent properties in the population.

Motivated by this observation, we develop a novel radial visualization scheme where the survivors are placed onto a two-dimensional plane with respect to their violence intensity and/or types. The objective of our approach is to present the interplay between the physical, psychological, and sexual components of violence in a visually accessible and comprehensible manner.

In order to visualize the survivors on a two-dimensional plane of violence type and intensity, we utilize a transformation scheme that is originally proposed for transforming the color space. This transformation (known as HSL) aims to represent a color on a three dimensional space having hue, saturation and luminance as axes instead of the usual red-green-blue (RGB) axes. In this space, hue indicates the color type (e.g., measures the difference between red and yellow colors), saturation indicates the color homogeneity (e.g., measures how much the color is different from gray), and the luminance indicates the brightness of the color (e.g., measures the difference between black and white).

In our case, when we consider that each of the three violence types corresponds to a different color (red, green and blue), the hue and luminance components of the HSL transformation essentially indicate the violence type and the intensity respectively. From a given set of rank normalized scores *r*_*PV*_ (*i*), *r*_*PA*_(*i*), and *r*_*SV*_ (*i*) for survivor *i*, we compute the HSL components as follows:^15^

(3) $$\begin{array}{l} I_{\max}(i)=\max\left\{r_{PV}(i),r_{PA}(i),r_{SV}(i)\right\},\;I_{\min}(i)=\min\{r_{PV}(i),r_{PA}(i),r_{SV}(i)\},\\ \;\;\text{Intensity}\;(i)=\left(I_{max}(i)+I_{min}(i)\right)/2. \end{array}$$

Now letting *M*(*i*) denote the violence type with maximum rank-normalized score for survivor i and setting Δ(*i*) = *I*_*max*_(*i*) − *I*_*min*_(*i*), we quantify the Type of violence for survivor *i* as follows:

(4) $$\begin{array}{l} H^{\prime }(i)=\{\begin{array}{ll} \;\text{undefined,}\; & \;\text{if}\;\Delta (i)=0,\\ \left(\left(r_{PA}(i)-r_{SV}(i)\right)/\Delta (i)\right)\;\mathrm{mod}\;6 & \;\text{if}\;M(i)=PV,\\ \left(\left(r_{SV}(i)-r_{PV}(i)\right)/\Delta (i)\right)+2 & \;\text{if}\;M(i)=PA,\\ \left(\left(r_{PV}(i)-r_{PA}(i)\right)\Delta (i)\right)+4 & \;\text{if}\;M(i)=SV. \end{array}\\ \;\;\mathrm{Type}(\text{i})=H^{\prime }(i)\times \pi /6 \end{array}$$

Note that Type indicates an angle, thus, it is defined in radians.

Using *Intensity* (corresponding to violence intensity) and *Type* (corresponding to violence type), we compute the location of survivor *i* in the two-dimensional plane as:

(5) x(i)=Intensity(i)×cos(Type(i)),y(i)=Intensity(i)×sin(Type(i))

The visualization of the survivors in this violence type vs. intensity space is shown in Figure 1, bottom right panel. As seen in the figure, in this space, the distance from the center ([0, 0]) indicates the intensity of violence, and the arc angle indicates the type of violence.

### 2.5. Clustering of Survivors and Identification of Subgroups

We apply clustering to identify subgroups of survivors based on their responses to the 39 items in the violence questionairre. For this purpose, we cluster the survivor profiles using K-means clustering with Euclidean distance by employing *kmeans* function in MATLAB Statistics and Machine Learning Toolbox.^16^ In order to find more reliable clusters, we run *K*-means 100 times and select the clustering with the minimum total within-cluster distance (sums of point-to-centroid distances). We use different values of *K* to optimize the number of clusters using Calinski Harabasz Evaluation.^17^

### 2.6. Health Problems and Trauma Symptoms

To investigate the relationship of reported violence with health problems and trauma symptoms, we compute category scores for health problems and trauma symptoms subscale as previously described for the violence subscales. Subsequently, we bin survivors according to their location in the violence intensity vs. violence type space and compute the average scores for health problems and trauma symptoms in each bin. We use radial visualization to visualize this results. This visualization allows the investigation of health problems and trauma symptoms with respect to violence type and intensities.

## 3. Results

### Individual item frequencies and assignment of items to violence types.

We first investigate the overall reporting frequency of individual items in the IPV questionnaire. The results of this analysis are shown in Figure 2. The bar plot in Figure 2(a) shows the frequencies of all 39 items, grouped by subscales (violence types). As seen in the figure, items that belong to the Psychological Aggression (PA) subscale are most frequently reported by survivors of violence, while items in the Sexual Violence (SV) subscale are reported least frequently.

The projection of the items to the two-dimensional principal component space is shown in Figure 2(b). As seen in the figure, items that belong to the same subscale are clustered in this reduced dimensional space. If we consider these two principal components as “eigensurvivors”, it is clear that these eigen-survivors tend to report similarly on all items (i.e., the items lie on a linear line with a positive slope), with the exception of PA3 (“called you names like ugly, fat, crazy, or stupid?”). This item lies as an outlier in the principal component space. Since this item is the most frequently reported item in the questionnaire and has a substantial influence on the PCA analysis, we decided to investigate it separately and labeled it as microaggression (MA). We also observe that PV1(“made threats to physically harm you?”) can be considered psychological aggression and lies close to psychological violence in the principal component space. Similarly, PV8 (“forced you to engage in sexual activity?”) involves sexual violence and lies close to SV items in this space. For these reasons, we move these items to the respective subscales.

### Co-occurrence of violence types.

**Fig. 3:**
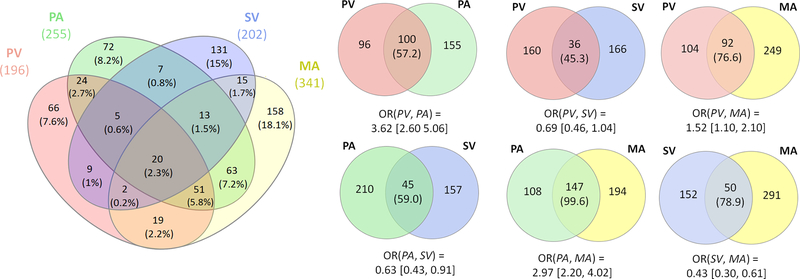
Co-occurrence of different types of intimate partner violence. The sets represent Physical Violence (PV, red), Psychological Aggression (PA, green), sexual violence (SV, blue), or Micro Aggression (MA, yellow). The first number in each set shows the number of survivors who report that type of violence above population mean. For the 4-way Venn-diagram, the numbers in parentheses show the percentage of survivors (over all survivors) in the respective set. The 2-way Venn diagrams assess the significance of the overlap between pairs of violence types, where the number in parenthesis shows the expected value of the intersection given the frequencies of each type. The resulting odds ratios (and 95% confidence intervals for the ORs) are shown below the Venn diagrams.

Once the assignment of items to violence types is finalized, we investigate the co-occurrence of violence types. The results of this analysis are shown in Figure 3. We observe significant co-occurrence of physical violence and psychological aggression, with an odds ratio of 3.62 (95% confidence interval: [2.60, 5.06]) and a linear correlation of 0.449 (*P*<0.001). While the co occurrence between physical violence and micro-aggression is weaker (OR=1.52, correlation=0.218, *P*<0.001), we observe that micro-aggression and psychological aggression occur frequently together (OR=2.97, correlation: 0.385, *P*<0.001). Interestingly, sexual violence tends to exhibit significantly less co-occurrence with all other types of violence, with an odds ratio below 1.0 and near zero (below 0.1) correlation for all other violence types (correlations: PV-SV=0.015, PA-SV=0.08, MA-SV=0.007). The 95% confidence intervals for the odds ratios fall completely below 1.0 for SV vs. PA and for SV vs. MA.

### Co-occurrence of individual items.

**Fig. 4:**
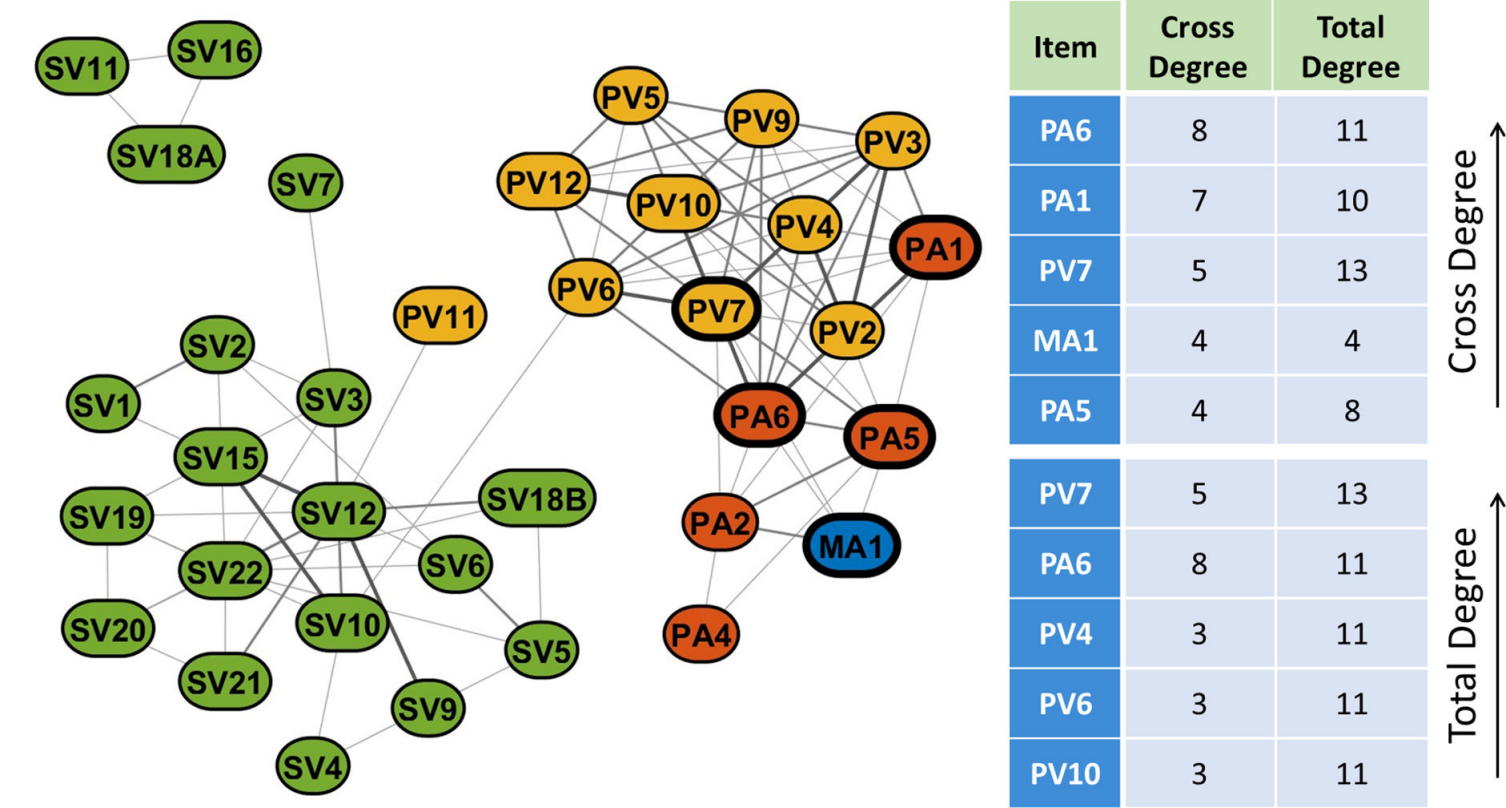
Itemwise co-occurrence network of intimate partner violence. The nodes represent violence items and the edges indicate the existence of positive correlation (> 0.2) between survivors’ responses to the corresponding pair of items. The widths of edges show the strength of correlation. The nodes (items) are colored according to their corresponding subscale: Yellow for Physical Violence (PV), red for Psychological Aggression (PA), green for Sexual Violence (SV), blue for Micro-Aggression (MA). The top five nodes with highest degree and highest cross-degree (with other violence types) are shown on the right.

To assess the co-occurrence patterns of IPV at a higher resolution, we also investigate the co-occurrence at the level of individual items. For this purpose, we assess the correlation between all pairs of the 39 items, and construct a network by retaining all pairs with correlation > +0.2. As seen in Figure 4, the network has two large connected components connected by a single weak edge.

One of these components represents Sexual Violence (SV), while Physical Violence (PV), Psychological Aggression (PA), and Micro-Aggression (MA) are together represented by a single component. We observe that the correlations among items within PV are stronger, with PV7 (“slammed you against something”) being the central node in the PV-PA cluster. The central item for the SV component, on the other hand, is SV12 (“used physical force or threats to physically harm you to make you have vaginal sex”). Interestingly, PV11 (“burned you on purpose ”) is also connected to SV12, although it is not connected to any other PV item.

### Clustering of survivors to identify violence subgroups.

**Fig. 5:**
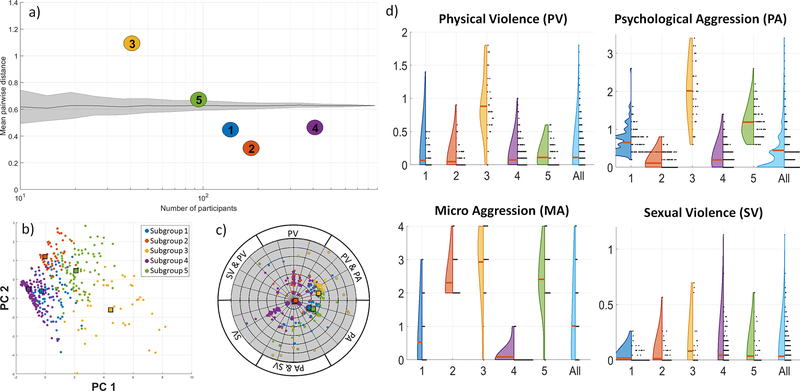
Clustering of survivors and the identification of subgroups. a) Size (number of survivors, log-scaled) vs. heterogeneity (measured by mean pairwise distance between survivors) of the five clusters of survivors identified using K-means. The black line and the grey area show the mean/95% confidence interval for the heterogeneity of random groups of survivors as a function of size (100 permutations). (b, c) Visualization of clusters in the two-dimensional principal component space/radial projection. Each survivor is colored according to their cluster/subgroup. Colored squares show the centers of respective subgroups. (d) Distribution of the scores for four different violence types in the identified subgroups.

To understand whether the survivors induce coherent subgroups in their reporting of violence and whether these subgroups are aligned with reported violence types, we use *K*-means to cluster the survivors. Using Calinski-Harabasz evaluation,^17^ we determine that *K* = 5 provides a reasonable balance between model fit and complexity. The resulting subgroups are shown in Figure 5.

We observe that subgroups with more survivors tend to be more homogeneous, where the smallest subgroup (#3) is significantly more heterogeneous than would be expected for a random group of survivors (Figure 5(a)). Visualization of the survivors in the subgroups in the two-dimensional principal component axes (Figure 5(b)) and radial axes (Figure 5(c)) shows that subgroups #2 and #5 are well-separated from other subgroups and this separation is reflective of the intensity of violence. In contrast, subgroups #1, #3, and #4 are separated from each other mostly based on the type of violence. Based on the distribution of the scores of violence types in each subgroup (Figure 5(d)), we annotate these subgroups as follows:

- *Subgroup #1:* Very low intensity of sexual violence, low/moderate intensity of other violence types.
- *Subgroup #2:* Very high intensity of micro-aggression, low intensity of psychological aggression, low/moderate intensity of other violence types.
- *Subgroup #3:* Very high intensity of all violence types.
- *Subgroup #4:* Variable intensity of sexual violence, low intensity of other violence types, particularly very low intensity of micro-aggression.
- *Subgroup #5:* Very high intensity of psychological aggression and micro-aggression, low intensity of physical violence and sexual violence.

### Health problems and trauma symptoms reported by survivors.

**Fig. 6:**
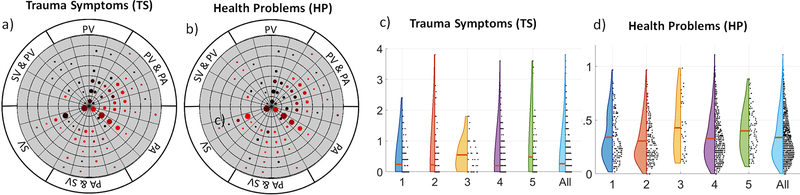
Relationship between IPV types/subgroups and health problems/trauma symptoms. (a)/(b) Radial visualization of trauma symptoms/health problems reported by the survivors. survivors are binned into small groups (shown as circles) based on their violence type and intensity. Distance from the center indicates the intensity of the violence, the angle indicates the type of violence. The size of the circle indicates the number of survivors in the corresponding group. The intensity of red indicates the prevalence of trauma symptoms/health problems reported by the survivors in that group. (c)/(d) The distribution of the prevalence of trauma symptoms and health problems reported by the survivors in each subgroup identified by clustering (see Figure 5).

In addition to the violence variables, NISVS also screens survivors for trauma symptoms and health problems. To understand how these health problems and trauma symptoms correlate with violence types and subgroups, we assess the distribution of survivors’ responses to these questions in the radial axis of PV-PA-SV, and in the subgroups we identify via clustering. The results of these analyses are shown in Figure 6. As seen in Figure 6(a), trauma symptoms are most commonly reported at the presence of intense psychological aggression and this effect is more pronounced when physical violence is also present. We also observe a similar pattern for health problems in Figure 6(b); however, health problems are also amplified with the presence of sexual violence. The distributions of these two variables in the five subgroups (Figure 6(c)/(d)) also show that trauma symptoms and health problem are most frequently reported in Subgroup #3, the subgroup that is associated with most intense psychological aggression and physical violence. The other subgroup that reports trauma symptoms and health problems above the population mean is Subgroup #5, which is associated with very high intensity of psychological aggression and micro-aggression, despite having lower levels of physical violence. We also observe that Subgroups #2 and #4 have long tails for trauma symptoms, while the long tail of the entire population for health problems is carried by Subgroup #4, indicating that very high intensity of sexual violence can be associated with significant health problems.

## 4. Discussion

Severity and type of violence perpetrated in the relationships have been increasingly utilized to understand patterns of IPV. In this study, we aimed to identify these patterns. Our results indicated that physical violence occurs frequently with psychological agression, its co-occurrence with micro-aggression is weaker (Figure 3). We also found that sexual violence tends to overlap less with all other types of violence. We also observed that individual items for sexual violence formed a single connected component in the co-occurrence network of individual items. This is one of the important findings of this study. It is important to note that the sexual violence in our analysis only includes acts of sexual violence perpetrated by intimate partners, as we restricted our analysis to instances in which the survivor is in an intimate relationship with the perpetrator.

Our network analysis indicated that the co-occurrence of physical violence items is more common compared to other types of violence (Figure 4). Slamming the partner against something exhibited strong co-occurrence with other physical violence items, as well as psychological aggression items. Other physical violence items with “high degree” included beating and hitting with a fist or something hard. Interestingly, these items were not as frequent as the most frequent physical violence items, such as slapping, pushing, and showing (Figure 2). Thus the presence of these moderate-frequency high-co-occurrence items may be indicative of more systemic physical violence. Making threats to harm the partner was more frequent, and also exhibited strong co-occurrence with many physical violence and psychological aggression items. Acting very angry toward the partner in a way that seemed dangerous almost exclusively co-occured with physical violence. The observation that the violence items that tend to co-occur with other items are not necessarily more prevalent suggests that these co-occurrence patterns can be useful in dissecting the etiology of violence in a relationship.

The sexual violence item that most frequently co-occured with other sexual violence items was “used physical force or threats to physically harm you to make you have vaginal sex”. The physical violence item “burned you on purpose” was also connected to sexual violence, although it was not connected to any other physical violence item.

Our data driven definition of micro-aggression is conceptually consistent with the widely used definition of micro-aggression. Although micro-agression is a relatively new construct and is still in the process of refinement, it draws considerable attention by researchers. Our findings can help application of this concept in relationships.

With cluster analysis, we identified five subgroups of intimate partner violence (Figure 5). These subgroups were mostly aligned with violence types, with micro-aggression claiming its own subgroup. The distribution of sexual violence in the subgroups was variable and seemed to exclude micro-aggression. An important outcome of cluster analysis was that severe psychological abuse seems to underlie two different forms of severe violence; one with intense micro-aggression and another with severe physical violence.

A longitudinal study investigating the mental health trajectories of IPV victims indicated that women who were exposed to psychological abuse were less likely to recover overtime from mental health issues such as depression, anxiety and PTSD.^18^ Past research also showed higher levels of mental health deterioration when both psychological and physical violence were co-occurring.^19^ Another study investigating court-involved battered women’s exposure to different types of IPV and the traumatic responses such as depression, acute stress and PTSD, demonstrated that they are associated while psychological abuse explained higher variance as compared to physical abuse.^20^ Intensity of the psychological, physical and sexual violence as well as the context and presence of one or more types of victimization is critical for our understanding to develop effective treatments.

In summary, it is crucial to understand the nature of the violence and develop strategies to effectively deliver treatments and support for the victims. Based on nationally representative data, we identified co-occurence patterns and subgroups of IPV. These results can be useful to develop screening tools as well as targeted and integrative treatment strategies.

## Figures and Tables

**Table 1:** The questionnaire items used in our study.

- **How many times did [perpetrator] … ?**
MA1: called you names like ugly, fat, crazy, or stupid
PA1: acted very angry towards you in a way that seemed dangerous
PA2: told you that you were a loser, a failure, or not good enough
PA4: insulted, humiliated, or made fun of you in front of others
PA5: told you that NO one else would want you
PA6: made threats to physically harm you

- **How many times did [perpetrator] … ?**
PV2: slapped you
PV3: pushed or shoved you
PV4: hit you with a fist or something hard
PV5: kicked you
PV6: hurt you by pulling your hair
PV7: slammed you against something
PV9: tried to hurt you by choking or suffocating
PV10: beaten you
PV11: burned you on purpose
PV12: used a knife or gun on you

- **How many times did [perpetrator] … you didn’t want it to happen?**
SV1: exposed their sexual body parts to you, flashed you, or masturbated in front of you
SV2: made you show your sexual body parts to them
SV3: made you look at or participate in sexual photos or movies
SV4: harassed you while you were in a public place in a way that made you feel unsafe
SV5: kissed you in a sexual way?
SV6: fondled or grabbed your sexual body parts
How many times did [perpetrator] … when you were drunk, high, drugged, or passed out and unable to consent?
SV7: had vaginal sex with you
SV9: made you receive anal sex
SV10: made you perform oral sex
SV11: made you receive oral sex

- **How many times did [perpetrator] used physical force or threats to physically harm you to make you … ?**
SV12: have vaginal sex
SV15: perform oral sex
SV16: receive oral sex
SV18a: (if male) try to make you have vaginal sex with them, but sex did not happen
SV18b: try to have (if female, vaginal) oral, or anal sex with you, but sex did not happen

- **How many people have you had vaginal, oral, or anal sex with after they pressured you by … ?**
SV19: doing things like telling you lies, making promises about the future they knew were untrue, threatening to end your relationship, or threatening to spread rumors about you
SV20: wearing you down by repeatedly asking for sex, or showing they were unhappy
SV21: using their influence or authority over you, for example, your boss or your teacher
SV22: forced you to engage in sexual activity
